# Revealing Charge Transfer Dynamics in Methylammonium Lead Bromide Perovskites via Transient Photoluminescence Characterization

**DOI:** 10.1021/acsaem.2c00561

**Published:** 2022-07-13

**Authors:** Jia Zhang, Jiajun Qin

**Affiliations:** † Biomolecular and Organic Electronics, IFM, Linköping University, Linköping 58183, Sweden; ‡ Department of Materials Science and Engineering, University of Tennessee, Knoxville, Tennessee 37996, United States

**Keywords:** perovskite grains, charge transfer, transient photoluminescence, excitonic emission, bimolecular recombination

## Abstract

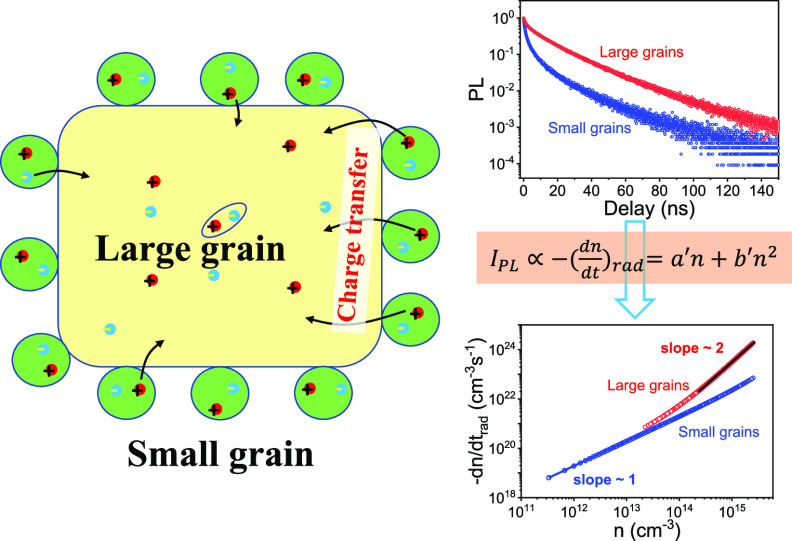

It is an important but difficult issue to identify charge and energy transfer processes in materials where multiple band gaps coexist. Conventional methods using transient absorption and optoelectrical characterization based on devices could not provide a clear picture of transfer dynamics. According to the bimolecular and monomolecular nature of each process, the carrier dynamics is supposed to solve this issue. In this work, we established a novel, convenient and universal strategy based on the calculation of carrier dynamics to distinguish energy/charge transfer and reveal their transfer dynamics in methylammonium lead bromide (MAPbBr_3_) films with mixing wide-band gap small grains and narrow-band gap large grains. A highly efficient charge transfer process is confirmed with a high negative nonradiative bimolecular recombination coefficient of −2.12 × 10^–7^ cm^–3^ s^–1^, indicating that free carriers within small grains are efficiently transferred from small grains to large grains. As a result, emission from large grains becomes dominant when increasing the photoexcitation intensity. In addition, current-density-dependent electroluminescence results in emission only from large grains, further verifying the charge transfer process. Moreover, it is interesting to find that when decreasing the size of small grains, the charge transfer process is facilitated, leading to an increased nonradiative bimolecular recombination coefficient from −2.12 × 10^–7^ to −4.01 × 10^–7^ cm^–3^ s^–1^ in large grains. Our work provides a convenient strategy to identify and quantify energy and charge transfer in metal halide perovskites, which can be used to enrich our understanding of perovskite photophysics.

## 1 Introduction

Toward a better understanding of the light emitting properties, it is highly desirable to reveal the carrier dynamics in the emerging materials like halide perovskites, which have become attractive candidates for new-generation lighting and display with low turn-on voltage, ambipolar transport, large color-tunability, and high device efficiencies.^1−8^ The polycrystalline nature which enables the combination of all kinds of crystallites, such as small/large 3D grains,^2^ 2D nanoplatelets,^9,10^ nanocrystals,^6^ and quantum dots,^11^ in the same perovskite thin films. The dynamical interactions such as energy/charge transfer^12^ in between these crystallites provide a new mechanism for improving various potential applications. For example, the energy funneling process between 2D nanoplatelets and 3D perovskite grains (to form quasi-2D perovskite films) leads to enhanced operational stability, improved light emitting property, and even novel stimulated emission with laser pulse doubling phenomenon.^9,13,14^ Since the dynamical interactions between different crystallites may vary with different film conditions, it is necessary to identify the carrier dynamical process to directly evaluate light-emitting properties of each individual film.

The carrier dynamics such as generation, transport, and recombination play a vital role in tremendous optoelectrical materials.^15−20^ Generally, carriers experience a wide range of time scales from subfemtosecond (for light absorption) to microsecond or even longer time [for photoluminescence (PL)].^21−25^ Thus, transient techniques have been developed to study the carrier dynamics, such as transient absorption (TA) and transient PL.^26−30^ However, the TA signal containing both photobleaching and photoinduced absorption signals cannot be directly correlated to the excitonic feature to reveal light-emitting dynamics.^31−33^ The conventional transient PL technique via detecting evolution of radiative recombination also experiences difficulty in extracting carrier dynamics due to complex relationship between PL and carrier density of the materials.^34−36^ Therefore, it requires a practical strategy to reveal the carrier dynamics.

Here, we proposed an experimental method to characterize carrier dynamics of perovskite films through combining transient PL and power-dependent PL results, where the time-evolution carrier density can be directly correlated with the PL intensity. Although similar attempts have been proposed through simulations,^37^ deviations may appear by simply applying an ideal model assuming that all the radiative recombination are from the bimolecular recombination. Here, we extend this model by considering that radiative recombination consists of both monomolecular recombination and bimolecular recombination in our MAPbBr_3_ perovskite films due to possible quantum confinement within nanometer sized subgrains. Through converting the PL intensity into carrier density via power-dependent PL results, transient PL results can be replotted into time evolution of the carrier density curve, that is, the carrier dynamics. As a practical case, perovskite films mixing small and large grains are studied based on our proposed method. It is found that charge transfer is a dominant process in the present perovskite films. Moreover, this conclusion is verified by the power-dependent steady-state PL and bias-dependent electroluminescence (EL) results. Furthermore, our method can also be applied to other perovskite films with a decreased size of small grains, where charge transfer is further facilitated and defects are passivated. Thus, we established a convenient and useful method to characterize the carrier transfer dynamics through transient PL results combined with power-dependent PL results.

## 2 Results and Discussion

### 2.1 Perovskite Film Mixed with Large/Small Grains

**Figure 1:**
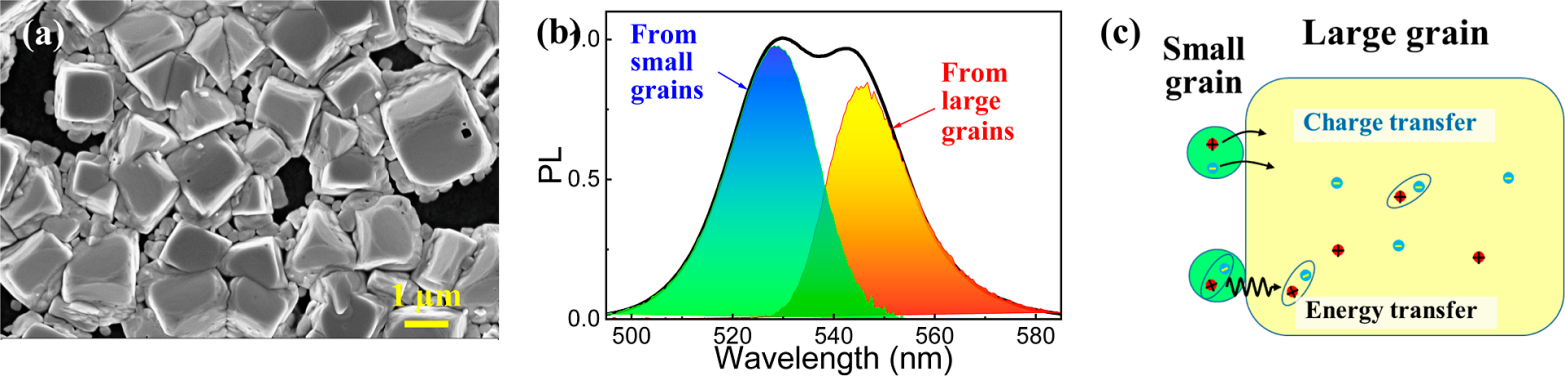
Identifications of the perovskite film with mixed large and small grains. (a) SEM images to show micrometer-size large grains attached by ∼100 nm-size small grains. (b) PL spectrum with two peaks; the left peak (blue and green colored) corresponds to emission from small grains, and the right peak (orange and red colored) corresponds to emission from large grains. (c) Schematic diagram to show two possible transfer processes between large and small grains: charge transfer and energy transfer.

The perovskite films are fabricated through one-step solution processing method, as reported previously.^2,38^ In the preparation of mixed large/small grain films, two solutions, one for preparing large grains (with lead bromide (PbBr_2_) and methylammonium bromide (MABr)) and one for preparing small grains (with lead acetate trihydrate (Pb(CH_3_CO_2_)_2_·3H_2_O) and MABr), are mixed together with the volume ratio of 4:1, resulting in the dominant precursors from the solution for preparing large grains. It should be pointed out that the PbBr_2_ is a strong electrolyte which can dissociate into free ions in the *N*,*N*-Dimethylformamide (DMF) solution thoroughly, while Pb(CH_3_CO_2_)_2_·3H_2_O is a weak electrolyte which can only partially dissociate into free ions. Thus, the perovskite crystallization rate of PbBr_2_ + MABr is much faster than that of Pb(CH_3_CO_2_)_2_·3H_2_O + MABr. During the film growth process, the crystallization from the reaction of PbBr_2_ and MABr will be dominant, leading to rapid formation of large grains initially. Afterward, the Pb(CH_3_CO_2_)_2_·3H_2_O + MABr source locates at the boundary of formed large grains to further crystallize into perovskites due to the slow crystallization rate. The follow-up perovskite crystal growth is slowed down and the partially dissociated CH_3_COO^–^ and H_2_O will easily terminate the grain growth, leading to gathered small grains. As shown in the scanning electron microscopy (SEM) image (Figure 1a), micrometer-size large grains are surrounded by the 100 nm-small grains. These two kinds of grains contribute to two individual peaks in the PL spectrum (Figure 1b) at around 528.5 and 546 nm, respectively. Since the composition of small and large grains are the same, the blueshifted PL peak at 528.5 nm from the small grains should originate from the quantum confinement effects, implying that the 100 nm-small grains contain even smaller subgrains with a size within the Bohr radius.

Specifically, the existence of perovskite nanostructures (small grains with the size smaller than the Bohr radius) is verified by the fact that increasing the amount of MABr in precursor solution would lead to significantly blueshifts of PL emission (Figure S1a). In addition, the PL emission at UV lamp illumination obviously changes its color from green to cyan when the molar ratio of Pb(CH_3_CO_2_)_2_·3H_2_O/MABr is varied from 1:2.4 to 1:4.5 (Figure S1b). Therefore, using the precursor solution with Pb(CH_3_CO_2_)_2_·3H_2_O and MABr will lead to quantum confined nanostructures. More detailed evidence on the coexistence of large and small grains can been found in our previous work.^2,38,39^ Similar grain size tuning by controlling the stochiometric ratio of MABr has also been reported in Lee’s group.^40^ Therefore, our fabrication method enables a very simple nanostructured perovskite film with different band gaps, which offers an ideal condition for searching the underlying optical and electrical physics in multiple band gap nanostructured perovskite LEDs.

Figure 1c shows the schematic diagram of possible processes under photoexcitation in this perovskite film. Essentially, both small grains (with a wide band gap of 2.35 eV) and large grains (with a narrow band gap of 2.27 eV) can generate excitons and emit light in the perovskite film. The energy offset (80 meV) enables excitons from small grains with a wide band gap to release the energy to large grains with a narrow band gap through the charge or energy transfer process. The charge transfer happens when dissociated electrons and holes from small-grain excitons funnel to lower-energy large grains. These electrons and holes recombine and consequently induce light emission in large grains. Owing to the recombination of free electrons and holes, charge transfer will lead to an increase in radiative bimolecular recombination. Contrarily, the energy transfer may also exist with the exciton energy directly transferred from small grains to large grains, leading to an increase in monomolecular recombination. Then, question arises that which process is dominant in our perovskite materials: charge transfer or energy transfer?

### 2.2 Carrier Dynamics Extracted from PL Lifetime Results

To answer the above question, we characterize carrier dynamics in both small and large grains through our proposed method. It should be noted that the carrier dynamics of a material can be expressed as
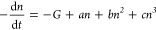
1where *n*, *t*, and *G* refer to carrier density, time and carrier generation rate, and *a*, *b* and *c* correspond to first-order, second-order, and third-order recombination coefficient.^41^ In this equation, both radiative and nonradiative recombinations are considered. In terms of radiative recombination (which leads to light emission) only, it is the combination of both first-order recombination and second-order recombination in hybrid perovskites. Therefore, the PL of a perovskite film can be interpreted as
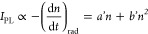
2where *a*′ and *b*′ refers to radiative monomolecular and radiative bimolecular recombination coefficient, respectively. It remains an open question on whether there is radiative monomolecular recombination at the surface, such as perovskite/organic, perovskite/perovskite, or perovskite/vacuum interfaces. Moreover, similar operation by considering both monomolecular and bimolecular radiative recombination paths in the 3D iodide-based perovskite films has already been proven to be successful in explaining the PL quantum yield results in different groups.^24,42^

Herein, we propose that the parameters *a*′ and *b*′ are achieved through power dependent PL results at low-excitation intensities based on the following analysis. In the case of steady state PL with continuous wave (CW) light excitation, rate equation of carrier density in eq 1 is in equilibrium (d*n*/d*t* = 0). Hence, *G* = *an* + *bn*^2^ + *cn*^3^. If the excitation intensity *P* (*P* ∝ *G*) is very low, only the first-order monomolecular recombination is dominant (i.e., *an* ≫ *bn*^2^ + *cn*^3^), leading to *P* ∝ *n*. As a consequence, eq 2 can be rewritten as
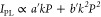
3

We should note that *k* is a scalable constant, and the equation is solid only when *P* is proportional to *n*. With eq 3, we can attribute the radiative recombination type of a material according to the logarithmic slope of the CW-power-dependent PL intensity, whereas the slopes that equal 1 and 2 refer to monomolecular-only and bimolecular-only radiative recombination, respectively. When *P* is increased, the second-order term (*bn*^2^) becomes not negligible as compared with the first-order term (*an*) due to increased carrier density, resulting in the deviated linear relationship between *P* and *n*. This is why we usually lose the linear tendency of the logarithmic power-dependent PL plot at higher CW excitation intensities.^43,44^ Therefore, we can only plot eq 2 and 3 by fitting the power-dependent PL intensity results on the condition that the CW excitation intensity is very low. After we settle the parameter *a*′ and *b*′ for radiative recombination, the transient PL curve can be directly converted to a transient carrier density curve (carrier dynamics) by eq 2. We can see more detailed procedures on how to convert the PL lifetime data into the carrier dynamics in the Supporting Information. To make it clear on the suitable CW excitation intensity region for our method, we simulate the *n*–*P* relation in Figure S2 with different *a* values. Obviously, the linear relationship of carrier density versus the CW excitation intensity occurs only at low excitation intensities, and this region may vary with different monomolecular recombination coefficients *a*. Typically, *a* is inverse proportional to the carrier lifetime, whereas ∼1 μs lifetime corresponds to *a* value of ∼10^6^ s^–1^.

### 2.3 Charge Transfer in Mixed Large/Small Grains

Now, we apply the above analysis to our perovskite films mixing with small and large grains. It should be noted that the trap filling process sometimes will cause the failure of our method due to variable trapping and detrapping rates with the excitation intensity. In this case, we can expect a prolonged PL lifetime if we increase the photoexcitation intensity. In the present system with mixed large/small grains, we can exclude the influence of trap-filling process because nearly unchanged PL lifetime curves at low excitation intensities were observed.

**Figure 2:**
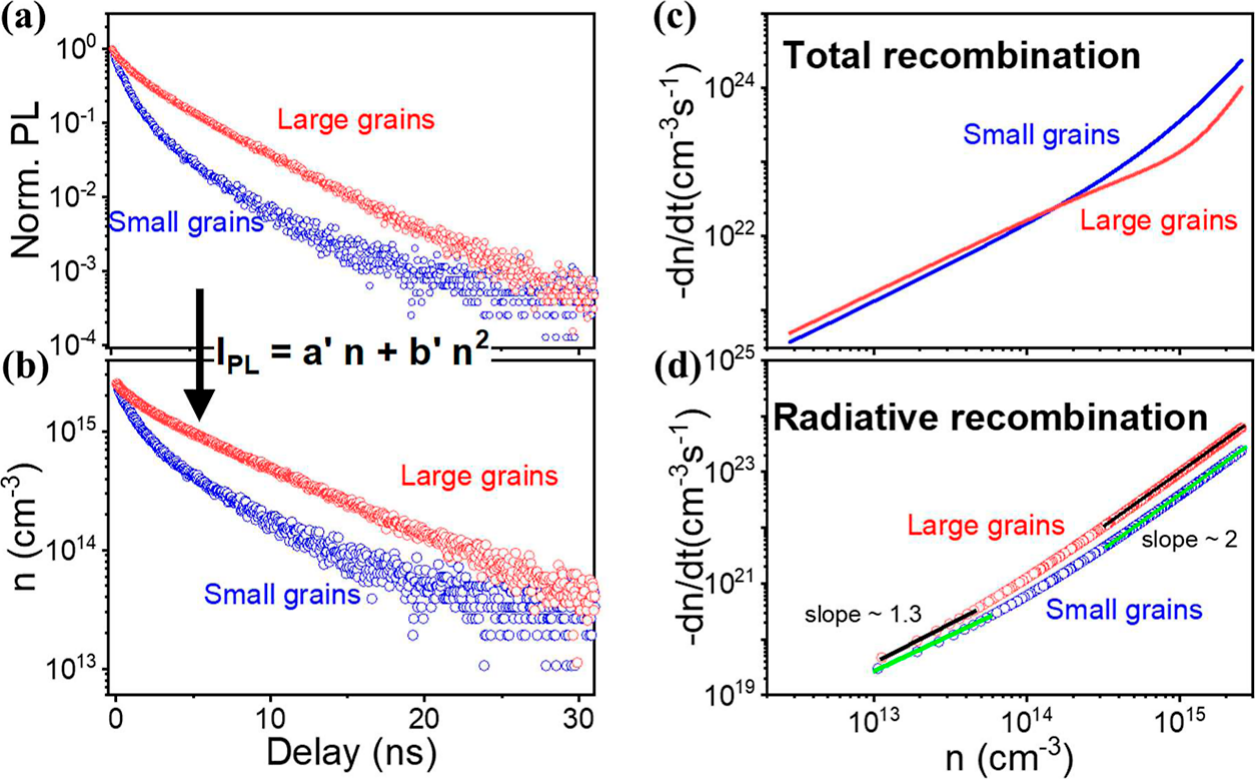
Carrier dynamics of small grains (blue curve) and large grains (red curve) deduced from transient PL results. (a) Transient PL results of small grains (detected at 505 nm) and large grains (detected at 545 nm). (b) Time evolution of carrier density extracted from (a). (c) Calculated total recombination rate as a consequence of carrier density. (d) Radiative recombination rate as a consequence of carrier density.

As shown in Figure 2a, two different emission wavelengths (505 and 545 nm) are detected to represent the transient PL results of small grains and large grains separately. Interestingly, the small grains experience a much faster decay initially in the time region from 0 to around 5 ns. After 10 ns, the decay slows down, showing slower decay as compared with large grains. By combining the power-dependent PL results in Figure S3, we can convert Figure 2a to 2b, which directly shows the time evolution of carrier density after pulsed laser excitation (femtosecond laser with a wavelength of 343 nm). Notably, details about the calculation of absolute initial carrier density at time *t* = 0 can be found in the experimental section by associating absorption results in Figure S6. Similar to the transient PL intensity, the carrier density of small grains decays faster initially and slows down after 10 ns as compared to that of large grains. After fitting with eq 1, recombination coefficients of both small and large grains are obtained (listed in Table 1). Here, we can derive *a* = 1.29 × 10^8^ s^–1^ (1.72 × 10^8^ s^–1^) and *b* = 1.7 × 10^–7^ cm^–3^ s^–1^ (−1.19 × 10^–7^ cm^–3^ s^–1^) for small (large) grains. As a result, total recombination rates of both small grains and large grains can be plotted as a function of carrier density (see Figure 2c). It agrees with Figure 2a,b that recombination rate of small grains is faster initially at a higher carrier density and slows down after decaying to a low carrier density as compared with large grains. By considering the PLQY value of ∼0.4% at CW excitation (100 mW/cm^2^), we can extract the radiative recombination rate (Figure 2d) from Figure 2c, with the fitted radiative/nonradiative recombination coefficients shown in Table 1. The logarithmic slope of both small grains and large grains shows a similar trend of ∼1.3 at a low carrier density (around 10^13^ cm^–3^) and ∼2 at a higher carrier density (above 5 × 10^14^ cm^–3^). It indicates that small and large grains possess both radiative monomolecular and bimolecular recombination even at carrier density as low as 10^13^ cm^–3^, corresponding to the case of 100 s mW/cm^2^ in CW excitation. Besides, the total recombination rate versus carrier density (Figure 2c) of both small and large grains keeps linear tendency (logarithmic slope = 1) at carrier density below 10^14^ cm^–3^, enabling the solidity of our fitting in Figure S3 (*P* < 100 mW/cm^2^, which fulfills the requirement that *P* ∝ *n*).

**Table 1:** Recombination Constants of Small and Large Grains in the Perovskite film^a^

		fitted parameters	
component	recombination	*a* (s^–1^)	*b* (cm^–3^ s^–1^)
small grains	total	(1.29 ± 0.07) × 10^8^	(1.7 ± 0.1) × 10^–7^
	radiative	(2.44 ± 0.03) × 10^6^	(3.60 ± 0.04) × 10^–8^
	nonradiative	(1.26 ± 0.08) × 10^8^	(1.33 ± 0.11) × 10^–7^
large grains	total	(1.72 ± 0.05) × 10^8^	(−1.19^b^±0.08) × 10^–7^
	radiative	(3.10 ± 0.03) × 10^6^	(9.38 ± 0.10) × 10^–8^
	nonradiative	(1.69 ± 0.06) × 10^8^	(−2.12 ± 0.13) × 10^–7^

a These parameters are extracted from transient PL results (Figure 2a,b).

b Negative value indicates the inverse process of carrier decrease, that is, transfer process with an increase in carrier density.

Our fittings are based on the rate equation where recombination processes (with positive *a*, *b*, *c* coefficients) refer to the decrease in carrier density. Thus, the negative recombination coefficients correspond to the increase in carrier density. In our system, the total bimolecular recombination coefficient of large grains is negative, indicating that the carriers are increased rather than decreased through the bimolecular pathway. We attribute the increase in carriers to the charge transfer process rather than the energy transfer process owing to the bimolecular electron hole recombination nature.

**Figure 3:**
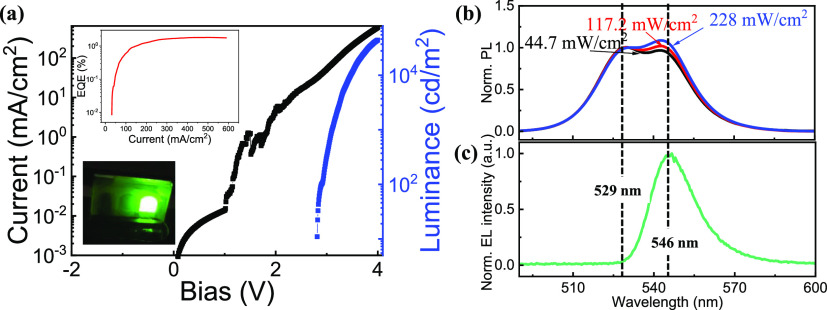
Comparison of EL and PL results to verify the charge transfer process between small and large grains. (a) Current density–voltage–brightness (*J*–*V*–*L*) curve of the perovskite LED based on the device structure of [ITO/PEDOT/PSS/MAPbBr_3_/Bphen/PMMA/LiF/Ag]. The inset is the EQE versus current density curve and the photo of the emission pixel. (b) PL spectra of the perovskite film under different photoexcitation intensities (CW laser, 405 nm): 44.7, 117.2, and 228 mW/cm^2^. (c) EL spectrum at bias = 2.7 V.

To verify the charge transfer process that occurs within our film, we compare the spectra under two excitation conditions: photoexcitation with initially generating electron–hole pairs/excitons (via PL) and electrical excitation (via EL) with initially generating separated free electrons and holes. The EL performance is measured with a perovskite LED device based on the device structure of ITO/PEDOT/PSS/perovskite/Bphen/PMMA/LiF/Ag (the schematic energy diagram is shown in Figure S4). This device shows good light emitting properties with a maximum EL brightness of 44,014 cd/m^2^ and maximum external quantum efficiency (EQE) of 1.8% in Figure 3a. Moreover, EQE can still maintain >1% when the current density is as high as 600 mA/cm^2^. The turn-on voltage of our device is around 2.9 V. As for the “jump” at around 1 V in the *I*–*V* curve, and we attributed it to the unexpected microconducting channels, which has been reported in our previous work.^45^ Interestingly, the EL emission spectrum in Figure 3c shows only one peak at 546 nm with a full width at half maximum (FWHM) of 19.3 nm. It indicates that only large grains emit light under the condition of carrier injection only. Here, if energy transfer occurs from small grains to large grains, electron–hole pairs should be formed in the small grains prior to transferring into the large grains. In this case, direct recombination to generate EL emission from small-grain component is expected due to the unignorable radiative recombination rate. However, in the real case, we cannot see any EL signal from small-grain component, verifying that charge transfer is the only transfer path. This conclusion is also consistent with the carrier dynamics analysis based on extracted carrier dynamics in Figure 2 and Table 1.

In EL, all injected carriers efficiently funnel to large grains, resulting in high EL brightness. In PL, photo-generated electron–hole pairs/excitons from small grains partially dissociate into carriers and funnel to large grains, contributing to the PL from large grains and consequently resulting in two PL peaks from both grains. When the photoexcitation intensity *P* increases from 44.7 mW/cm^2^ to 228 mW/cm^2^, the relative PL intensity (PL intensity from large grains relative to that from small grains) increases 12%, indicating that more carriers from small grains funnel to large grains (in Figure 3b). This result further verifies the fact that the charge transfer is a dominant process in our perovskite LEDs.

Ultrafast TA results are also introduced based on the same perovskite film to show the fast charge transfer process. As shown in Figure S5, TA spectra at different time delays and the TA dynamics detected at two different wavelengths indicate that the signal at a shorter wavelength (from 480 to 520 nm, corresponding to a small-grain component) decays faster than that at a longer wavelength (corresponding to a large-grain component). These results also confirm the charge transfer process from small grains to large grains.

### 2.4 Strategy to Facilitate Charge Transfer

**Figure 4:**
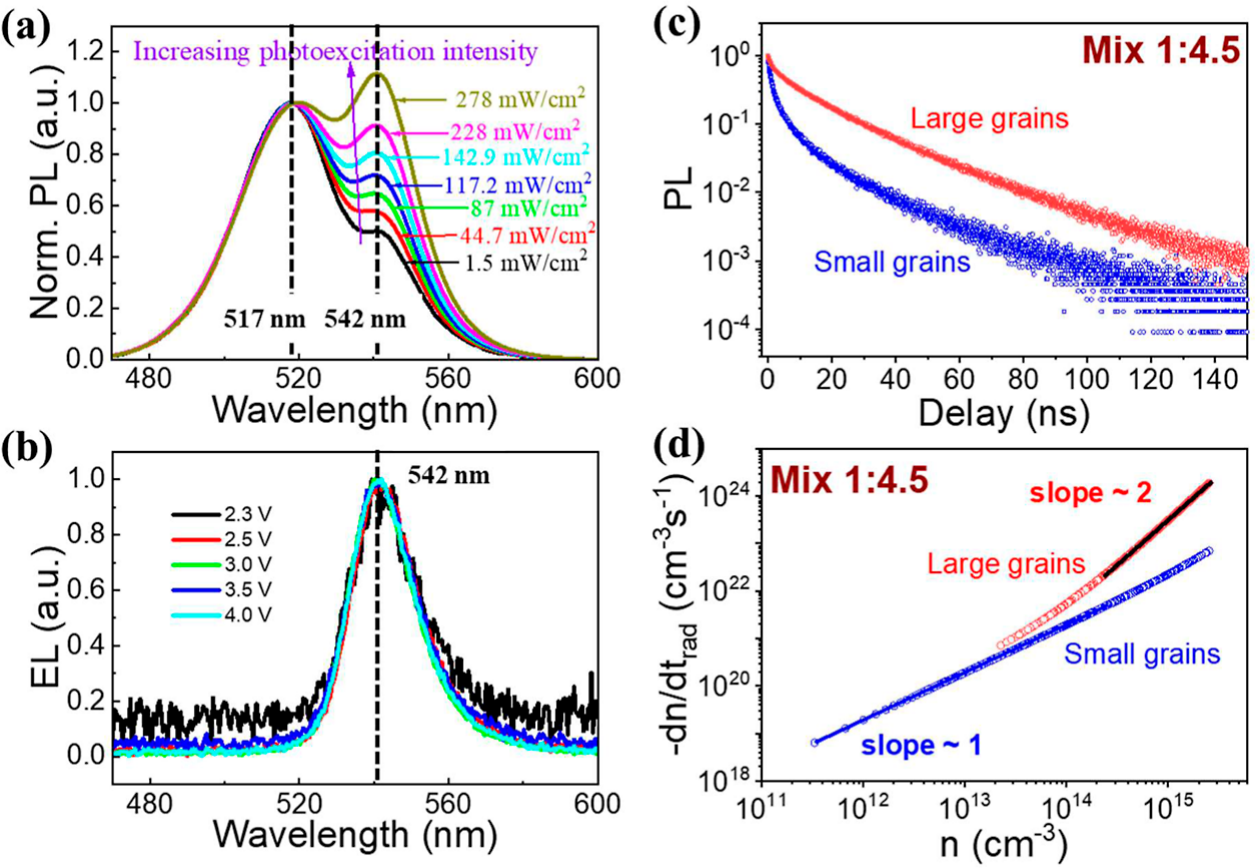
Experimental results to show two effects of the decreasing size of small grains in the perovskite film with mixed large/small grains: the passivating defect and facilitating charge transfer. (a) PL spectra of the perovskite film under different photoexcitation intensities (CW laser, 405 nm). (b) EL spectra at different biases (from 2.3 to 2.7 V). (c) Transient PL results of the perovskite film with excess MABr. (d) Radiative recombination rate of small grains and large grains as a consequence of carrier density.

During the film preparation, we found that the grain size of small grains can be decreased with an increasing amount of MABr in the precursor solution (see Figure S1). As shown in Figure 4a, by increasing MABr, the size of small grains can be decreased, resulting in the PL peak being shifted to 517 nm (which is 529 nm in Figures 1b and 3c). Simultaneously, the PL peak for large grains slightly shifts to around 542 nm. The optical band gaps extracted through the PL peak of small and large grains both increase with the grain size decreasing compared with the control sample shown in Figure 3c. We should note that PL emission from large grains increases more significantly with an increasing light intensity in the perovskite film with excess MABr (in Figure 4a) as compared with the control film in Figure 3b. To better clarify this difference, we normalize all the peaks from small grains to 1 in order to figure out the contribution of the photoexcitation intensity to the charge transfer efficiency. It is shown that in Figure 4a, the relative peak value at 542 nm (corresponds to a large-grain-component) increases from 0.571 at 44.7 mW/cm^2^ to 0.907 at 228 mW/cm^2^, showing an enhancement 59%. By contrast, the control film without excess MABr (in Figure 3b) shows a relative increment of only 12% (peak value at 543 nm, from 0.97 at 44.7 to 1.087 at 228 mW/cm^2^). Here, the relative values of large-grain-component emission are smaller in the film with excess MABr, which is attributed to the increased radiative monomolecular recombination in small grains due to a stronger quantum confinement (also consistent with Figure S1). The light intensity enhanced increase in large-grain-component provides an indication of the charge transfer process from small grains to large grains. Moreover, it also reveals that with the energy offset increase (from 75 meV in Figure 3b to 100 meV in Figure 4a) controlled by tuning grain sizes, the charge transfer becomes more efficient. By increasing the bias from 2.3 to 4.0 V based on the film with excess MABr, the EL spectrum in Figure 4b always fixes at 542 nm (corresponding to large-grain-only emission), which further indicates the dominant process of charge transfer.

To reveal this enhanced charge transfer process in the perovskite film with excess MABr, we characterize the carrier dynamics according to transient PL results (shown in Figure 4c). Much prolonged PL lifetimes of both small and large grains are observed, indicating passivation effects of MABr. Especially comparing the fitted parameters of Figure 2a (corresponding to Table 1) and Figure 4c (corresponding to Table S1), we can see that the total monomolecular recombination coefficient of the large-grain component is largely decreased from 1.72 × 10^8^ to 7.2 × 10^7^ s^–1^, while the total bimolecular recombination coefficient keeps nearly unchanged. This result indicates that the prolonged PL lifetime, which is a signature of a decreased total recombination rate, actually originates from the decrease in the monomolecular recombination coefficient. Hence, the defect passivation is the main contribution to the prolonged PL lifetime. Moreover, the radiative bimolecular recombination coefficient *b*′ of large grains is largely increased from 9.38 × 10^–8^ to 2.84 × 10^–7^ cm^–3^ s^–1^, indicating a more dominant free electron hole recombination owing to the facilitated charge transfer. It should be noted that, our dynamical analysis also demonstrates that the small grains tend to be excitonic-emission-dominant due to the increased first order contribution within the radiative recombination. The carrier-density-dependent radiative recombination curve in Figure 4d further verifies our analysis with the logarithmic slope near 1 even at a carrier density as high as 5 × 10^15^ cm^–3^. The increased monomolecular radiative recombination in small grains may originate from the increase in exciton binding energy due to a decreased grain size and eventually leads to a more dominant emission at a relatively low CW excitation intensity (1.5 mW/cm^2^ as an example) in Figure 4a as compared with large grains. Therefore, our carrier dynamics characterizations and the fitted parameters match perfectly with our experimental data such as PL and EL.

## 3 Conclusions

In summary, we proposed a method to characterize carrier dynamics of perovskite films by considering both monomolecular and bimolecular radiative recombination within the perovskite films. Combining power-dependent PL results and transient PL results, all the parameters in the rate equation can be obtained. With the carrier dynamics results, we found that a very efficient charge transfer occurs between small and large perovskite grains. Our PL results with two individual peaks from small and large grains, respectively, and EL results with only one peak from large grains, verifying this charge transfer process. Moreover, after adding excess MABr, the size of the small grains can be decreased, and facilitated charge transfer together with defect passivation can be revealed through carrier dynamics analysis and PL/EL spectra. Therefore, our proposed method to characterize transfer dynamics provides a convenient strategy to identify and predict the light emitting properties of halide perovskites. Furthermore, the applicability of our method can be also extended to other light-emitting materials such as organics or inorganics.

## 4 Materials and Methods

### 4.1 Materials Processing and Device Fabrication

The ITO substrates were first cleaned by ultrasonic treatment respectively with detergent, deionized water, acetone and isopropanol for 20 min in each cycle. The cleaned ITO substrates were exposed to UV ozone for 30 min and followed by the spin coating of PEDOT/PSS with the thickness of ∼40 nm. The PEDOT/PSS films coated on ITO substrates were annealed at 150 °C for 0.5 h.

The perovskite (MAPbBr_3_) films with mixed large/small grains were prepared based on the following procedures. First, two solutions were prepared: (i) 0.94 mol/L DMF solution (with a molar ratio of Pb(CH_3_CO_2_)_2_·3H_2_O/MABr = 1:3 for the unpassivated film and 1:4.5 for the film with excess MABr) for small grains and (ii) 1.58 mol/L DMF solution (with molar ratio PbBr_2_/MABr = 1:1.05) for large grains. Second, these two solutions with a volume ratio of 1:4 (more PbBr_2_ portion) were mixed to prepare the mixed large/small grains. The mixed solution was spin-coated onto PEDOT/PSS films under a nitrogen atmosphere at the rate of 3000 rpm to form MAPbBr_3_ films with mixed large/small grains and then annealed for 30 min at 60 °C.

The electron transport layer was spincast at the rate of 8k rpm for 60 s with the mixed chloroform solutions of Bphen (20 mg/mL) and PMMA (20 mg/mL) with a volume ratio of 1:2. Then, LiF and Ag were thermally deposited under vacuum with the thicknesses of 0.7 and 80 nm. Finally, the perovskite light-emitting devices were prepared with the architecture of ITO/PEDOT/PSS/MAPbBr_3_/Bphen/PMMA/LiF/Ag. The thicknesses of ITO, perovskite, and Bphen/PMMA layers are around 140, 700, and 100 nm, respectively. The fabricated perovskite light-emitting devices were encapsulated by UV-curable epoxy for experimental measurements.

### 4.2 Characterizations and Measurements

The film morphologies were characterized by SEM (Zeiss). The transient and steady-state PL characteristics were measured by using a Flouro Log III spectrometer with lifetime acquisition. For transient PL, we use a femtosecond laser with a wavelength at 343 nm, a repetition frequency of 200 kHz, and a spot size around 300 μm in diameter. The CW laser is 405 nm semiconductor laser with a spot size around 1 mm^2^. The excitation intensity is controlled by neutral density filters. The current–voltage characteristics were measured by Keithley 2400. For the EL characterization, a power meter (ST86LA) is introduced to characterize the EL brightness (in unit of cd/cm^2^). Simultaneously, the *I*–*V* curve is recorded for the calculation of the current efficiency. The conversion relationship between the current efficiency and EQE can be obtained by analyzing the EL spectrum (obtained by a Flouro Log III spectrometer), according to the literature.^46,47^

Carrier density under femtosecond laser excitation: according to eq 1, the carrier lifetime in our perovskite films is usually much longer than 150 fs, which is the time of one pulse of our transient light source. Hence, during one pump pulse, we can treat it as a net carrier density gain by absorption. Thus, we can get . Here, *n* refers to the carrier density. *R*, α, and *d* represent the reflectance, absorption coefficient, and film thickness, which are related to the absorption data in Figure S6. All the perovskite film thicknesses are ∼700 nm. *S*, *c*, *h*, *f*, *I*, and λ represent area of the excitation spot, the speed of light, Planck’s constant, pulse repetition frequency, average pump power, and excitation wavelength.

## Supporting Information Available

Detailed procedures to get the carrier dynamics through combining CW–PL data and PL lifetime data, (PDF)
