# Supplementary material for: Revealing Charge Transfer Dynamics in Methylammonium Lead Bromide Perovskites via Transient Photoluminescence Characterization
Source: ACS Appl Energy Mater. 2022 Jul 13;5(7):8084–91. doi: 10.1021/acsaem.2c00561 (PMC9344379; doi:10.1021/acsaem.2c00561)
Supplement: Supplementary file 1 — ae2c00561_si_001.pdf [file ae2c00561_si_001.pdf]

Supporting Information

Revealing charge transfer dynamics in methylammonium lead bromide perovskites via transient photoluminescence characterization

Jia Zhang[1,2,3] and Jiajun Qin[3]*

1 Wuhan National Laboratory for Optoelectronics, Huazhong University of Science and Technology, Wuhan, 430074, China

2 Department of Materials Science and Engineering, University of Tennessee, Knoxville, Tennessee 37996, USA

3 Biomolecular and Organic Electronics, IFM, Linköping University, Linköping, 58183, Sweden

*Corresponding Author:

Dr. Jiajun Qin: email: Jiajun.qin@liu.se

Detailed procedures to get the carrier dynamics through combining CW-PL data and PL lifetime data:

With the CW-PL data (PL intensity I versus excitation intensity P), we do the fitting with formula of $I = AP + BP^2$, where A and B are fitting parameters. By combining Eq. 2 in the main text and $P = kn$, we can get

$$I = Akn + Bk^2n^2 \tag{S1}$$

This relationship is also applied to the transient PL case by adjusting the variable constant k. Since we can get the n value at $t = 0$ (i.e. n_0 can be obtained by considering the light absorption in the experimental section), k can be calculated by solving Eq. S1. With the A, B, k in hand, the $n \sim t$ relationship can be extracted by representing the I-t curve in the PL lifetime results. In this case, the relationship of $-\frac{dn}{dt} \sim t$ can be induced simply to get the recombination coefficients a and b. When the PLQE value at fixed CW excitation intensity P (the corresponding carrier density can be calculated with the rate equation) is measured, we can get the values a' and b' by using the equation $PLQE = \frac{a'n + b'n^2}{an + bn^2}$.

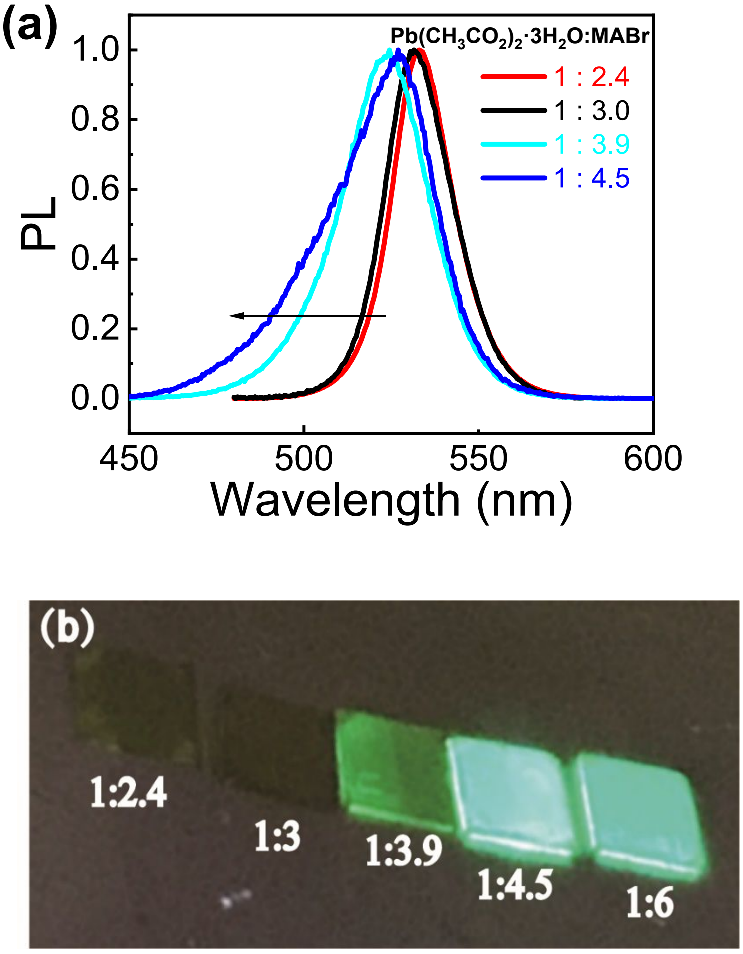

Figure S1. (a) Normalized PL spectra of perovskite films prepared with different stochiometric molar ratios of $Pb(CH_3CO_2)_2{\cdot}3H_2O$:MABr. (b) Photo of the corresponding films under UV lamp illumination.

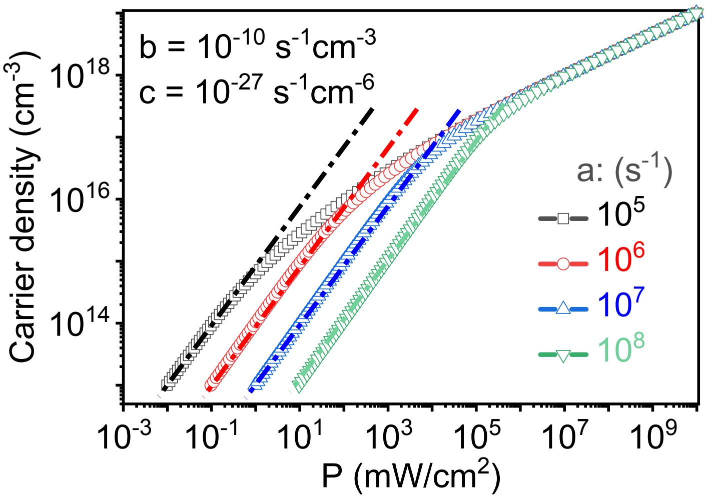

Figure S2. Carrier density values at different CW excitation intensities with different monomolecular recombination coefficients *a*: 10^5 s^{-1} (black), 10^6 s^{-1} (red), 10^7 s^{-1} (blue), 10^8 s^{-1} (green). Here, we set the film thickness of 100 nm, the absorption coefficient of 10^5 cm^{-1}, the reflectance of 0.2, and the CW excitation wavelength of 405 nm.

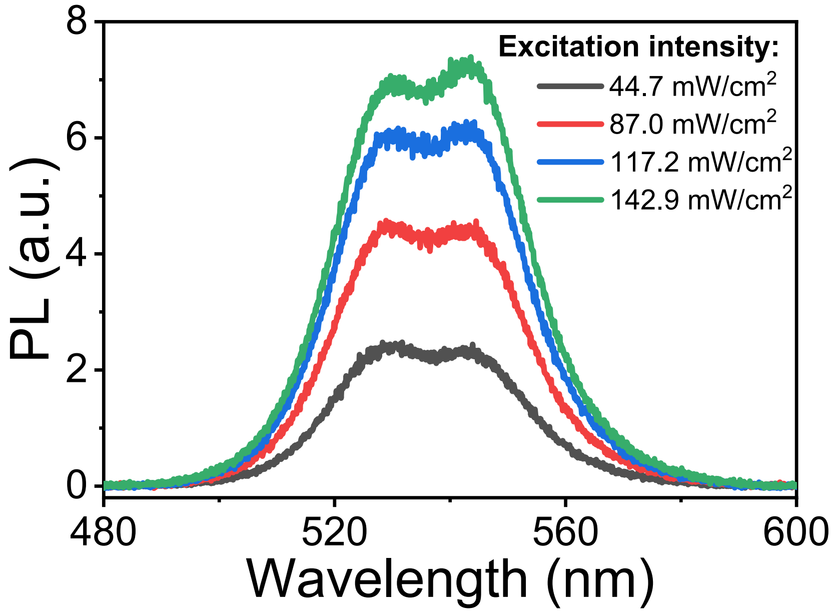

Figure S3. Power dependent PL spectra for perovskite film mixing small and large grains under CW excitation. The data is for the fitting of equation (2).

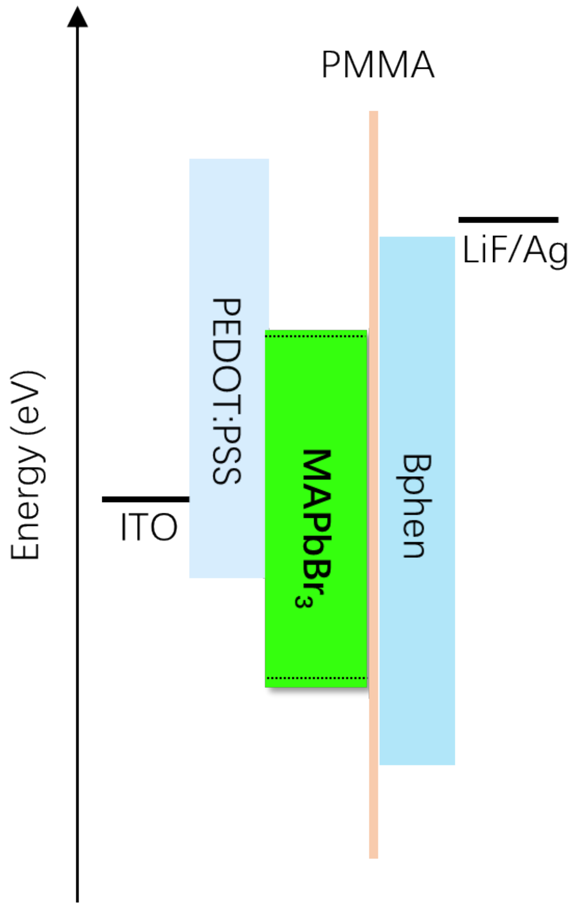

Figure S4. Schematic energy diagram of our perovskite device structure.

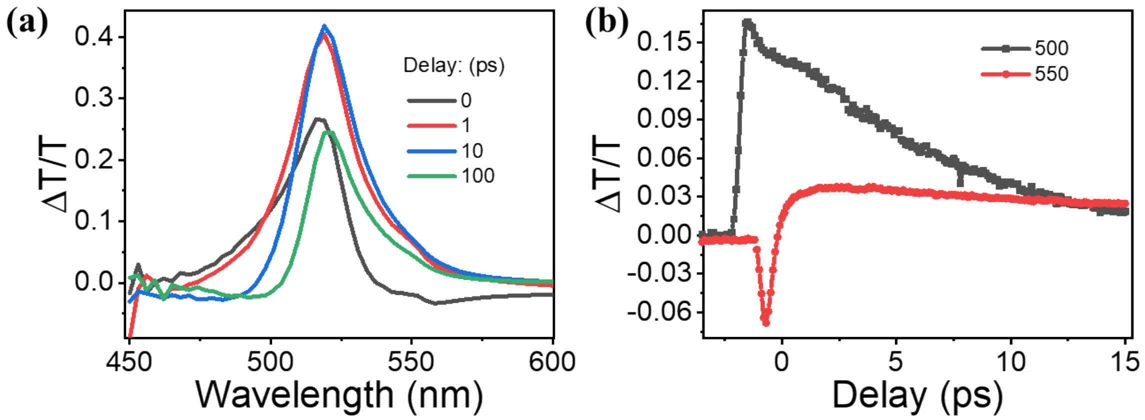

Figure S5. Transient absorption (TA) results. **(a)** TA spectra at different time delays: 0 ps (black), 1 ps (red), 10 ps (blue), and 100 ps (green). **(b)** TA dynamics detected at two different wavelengths: 500 nm (black, corresponding to small-grain component) and 550 nm (red, corresponding to large-grain component).

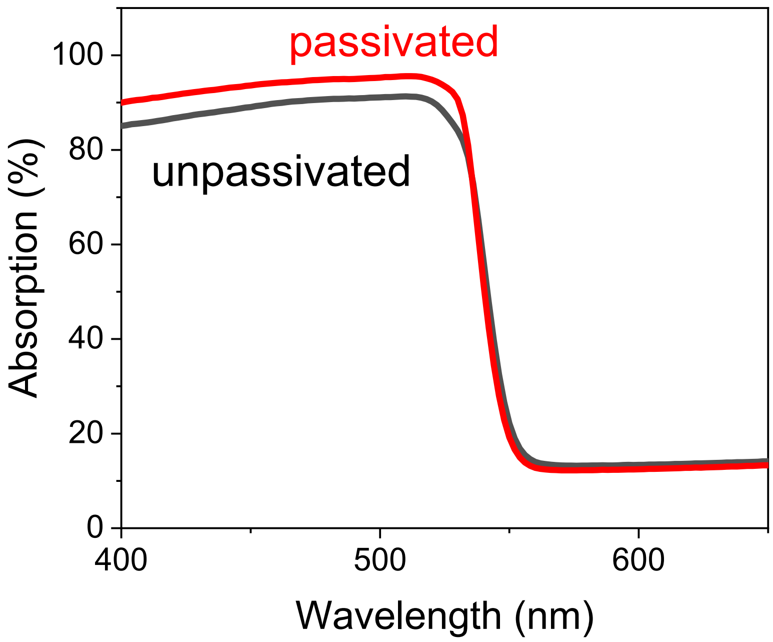

Figure S6. Absorption spectra of unpassivated film (correspond to Figure 1, 2 and 3) and passivated film (correspond to Figure 4).

Table S1. Recombination constants of small and large grains in perovskite film with excess MABr. These parameters are extracted from transient PL results (Figure 4a and b).

Component	Recombination	Fitted parameters	
		a (s^{-1})	b ($cm^{-3}s^{-1}$)
Small grains	total	(4.6±0.4) × 10^{7}	(2.40±0.06) × 10^{-7}
	radiative	(1.91±0.02) × 10^{7}	(3.43±0.03) × 10^{-9}
	nonradiative	(2.67±0.4) × 10^{7}	(2.37±0.07) × 10^{-7}
Large grains	total	(7.2±0.3) × 10^{7}	(-1.17±0.05) × 10^{-7}
	radiative	(2.56±0.03) × 10^{7}	(2.84±0.03) × 10^{-7}
	nonradiative	(4.64±0.03) × 10^{7}	(-4.01±0.06) × 10^{-7}
